# Mesenchymal Stem Cells from Human Exfoliated Deciduous Teeth and the Orbicularis Oris Muscle: How Do They Behave When Exposed to a Proinflammatory Stimulus?

**DOI:** 10.1155/2020/3670412

**Published:** 2020-02-25

**Authors:** Alessander Leyendecker Junior, Carla Cristina Gomes Pinheiro, Daniela Yukie Sakai Tanikawa, José Ricardo Muniz Ferreira, Mariane Tami Amano, Daniela Franco Bueno

**Affiliations:** Hospital Sírio Libanês, São Paulo, SP, Brazil

## Abstract

Mesenchymal stem cells (MSCs) have been studied as a promising type of stem cell for use in cell therapies because of their ability to regulate the immune response. Although they are classically isolated from the bone marrow, many studies have sought to isolate MSCs from noninvasive sources. The objective of this study was to evaluate how MSCs isolated from the dental pulp of human exfoliated deciduous teeth (SHED) and fragments of the orbicularis oris muscle (OOMDSCs) behave when treated with an inflammatory IFN-*γ* stimulus, specifically regarding their proliferative, osteogenic, and immunomodulatory potentials. The results demonstrated that the proliferation of SHED and OOMDSCs was inhibited by the addition of IFN-*γ* to their culture medium and that treatment with IFN-*γ* at higher concentrations resulted in a greater inhibition of the proliferation of these cells than treatment with IFN-*γ* at lower concentrations. SHED and OOMDSCs maintained their osteogenic differentiation potential after stimulation with IFN-*γ*. Additionally, SHED and OOMDSCs have been shown to have low immunogenicity because they lack expression of HLA-DR and costimulatory molecules such as CD40, CD80, and CD86 before and after IFN-*γ* treatment. Last, SHED and OOMDSCs expressed the immunoregulatory molecule HLA-G, and the expression of this antigen increased after IFN-*γ* treatment. In particular, an increase in intracellular HLA-G expression was observed. The results obtained suggest that SHED and OOMDSCs lack immunogenicity and have immunomodulatory properties that are enhanced when they undergo inflammatory stimulation with IFN-*γ*, which opens new perspectives for the therapeutic use of these cells.

## 1. Introduction

Mesenchymal stem cells (MSCs) are multipotent cells that have the ability to differentiate into mesodermal cell lineages, including chondroblasts, osteoblasts, and adipocytes [1, 2]. In addition, three basic characteristics must be present to classify a culture of cells isolated from neonatal or adult tissues as a culture of MSCs [3]. First, MSCs are able to adhere to the plastic of the cell culture flasks. Furthermore, at least 95% of an isolated and cultured cell population must express mesenchymal antigens (such as CD29, CD44, CD73, CD90, and CD105) and should not express hematopoietic or immune cell markers (such as CD14, CD19, and CD34) or endothelial cell markers (such as CD31). Finally, MSCs should be able to differentiate into osteoblasts, chondrocytes, and adipocytes in vitro under specific culture conditions [3].

Although they are more commonly isolated from the bone marrow and adipose tissue of donors, recent studies have demonstrated that MSCs can also be obtained from several other tissue types [4, 5] and that the cells from distinct tissues present considerable differences in their proliferative abilities and differentiation potentials [6]. For example, it has been suggested that MSCs obtained from neonatal tissues are more proliferative, have a higher differentiation potential, and can be maintained in culture for longer periods (before reaching cell senescence) than MSCs isolated from the bone marrow [6]. In addition, a study conducted by Barlow et al. [7] demonstrated that MSCs isolated from the human placenta have a high proliferative capacity and are able to integrate into tissues to be regenerated. Additionally, a study conducted by Kern at et al. [8] showed that MSCs isolated from adipose tissue also have a proliferative capacity superior to that of MSCs isolated from the bone marrow. Furthermore, this study demonstrated that MSCs isolated from adipose tissue have a high adipogenic potential and are able to be cultured for long periods without undergoing senescence or a loss of phenotypic characteristics [8]. Another type of MSC that is currently being investigated due to its potential in tissue engineering applications is stem cells from human exfoliated deciduous teeth (SHED). This type of stem cell has shown great capacities to differentiate into neural precursor cells, odontoblasts, and osteoblasts and has a high proliferative capacity [9]. In addition, Bueno et al. [10] demonstrated that MSCs isolated from the orbicularis oris muscle (OOMDSCs) obtained during cheiloplasty surgery in cleft lip and palate patients are capable of differentiation into chondrogenic, adipogenic, osteogenic, and skeletal muscle cells and present phenotypic and behavioral characteristics similar to those of MSCs isolated from other tissues. Due to their potential to differentiate into multiple cell types, MSCs could be used for the treatment of several diseases, especially for the repair of tissue lesions.

Many of the therapeutic properties of MSCs have also been attributed to the paracrine and endocrine effects of their secreted factors. Notably, MSCs have been shown to be capable of supporting the maturation and proliferation of hematopoietic cells, migrating to an area of tissue injury, recruiting tissue-specific progenitor cells [11], and regulating the immune response through the secretion of immunomodulatory cytokines and microvesicles containing a variety of bioactive molecules such as enzymes, coding and noncoding RNAs, and heat shock proteins [12]. In addition to the minimum criteria for the characterization of MSCs defined by the International Society for Cell Therapy [3], it has been proposed that the immunological properties of MSCs should also be used as one of the characterization criteria for MSCs [13]. The positive results in preclinical trials and demonstration of the immunomodulatory effects of MSCs in in vitro and animal studies have led to a rapid increase in the number of clinical trials in which the therapeutic potential of these cells has been evaluated for the treatment of a variety of diseases [14]. As a result, a large number of distinct cell preparations of MSCs have been tested in nearly 350 clinical trials conducted by academic institutions and corporations in which the safety and efficacy of autologous and allogeneic MSCs have been evaluated. Examples of diseases in which the therapeutic potential of MSCs has been evaluated in clinical and preclinical trials and has presented promising results include acute myocardial infarction [15], graft-versus-host disease [16], systemic lupus erythematosus [17], rheumatoid arthritis [17], Crohn's disease [18], multiple sclerosis [19], amyotrophic lateral sclerosis [20], and type I diabetes mellitus [21].

Although IFN-*γ* is a proinflammatory cytokine, studies have shown that IFN-*γ* also influences the osteogenic potential of MSCs. Croes et al. [22] demonstrated that activated CD4^+^ T lymphocytes cocultured with human MSCs promote the differentiation of the MSCs into osteoblasts, and after blocking secreted IFN-*γ* with antibodies, osteogenic differentiation of the MSCs was inhibited. In addition, a study conducted by Duque et al. [23] demonstrated that human MSCs secrete IFN-*γ* that acts by stimulating the osteogenic differentiation potential of the MSCs through the expression of osteogenic transcription factors, such as Runx2. Furthermore, a study conducted by Vidal et al. [24] demonstrated that MSCs isolated from mice with knocked-out IFN-*γ* receptors (IFN-*γ* R ^−^/^−^) express Runx2 at lower levels than MSCs isolated from wild-type mice and, therefore, have a more limited potential for osteogenic differentiation. In a study conducted by Liu et al. [25], it was demonstrated that MSCs isolated from the bone marrow had their potential for osteogenic differentiation inhibited when treated with 200 ng/mL IFN-*γ* compared with no stimulation with IFN-*γ*. However, it was also demonstrated that treatment of MSCs with IFN-*γ* at a concentration of 50 ng/mL had no inhibitory effect on the osteogenic differentiation potential of the MSCs [25]. This difference was attributed to the increased expression of SMAD6 (a gene that inhibits osteogenic differentiation) and decreased expression of Runx2, osteocalcin, and alkaline phosphatase in the MSCs treated with the highest IFN-*γ* concentration, whereas the expression of these genes remained unchanged in the MSCs treated with IFN-*γ* at a 50 ng/mL concentration [25]. Additionally, a study conducted by Sonoda et al. [26] demonstrated that dental pulp stem cells isolated from teeth with irreversible pulpitis and treated with IFN-*γ* at a 100 ng/mL concentration were able to give rise to a significant number of nodules containing calcium deposits (positive for Alizarin Red staining) after 4 weeks of culture in osteogenic differentiation medium. However, this same study demonstrated that dental pulp stem cells isolated from teeth with irreversible pulpitis that were not previously treated with IFN-*γ* gave rise to a much smaller number of nodules containing calcium deposits after 4 weeks of culture in osteogenic differentiation medium.

Regarding their immunomodulatory potential, MSCs, when exposed to a proinflammatory stimulus, will secrete molecules that act by inhibiting the maturation of antigen-presenting cells such as monocytes, dendritic cells (DCs), and macrophages. These molecules also promote the polarization of macrophages into M2 macrophages and inhibit the polarization of macrophages into M1 macrophages. MSCs are also able to inhibit the activation and proliferation of natural killer (NK) cells, CD8^+^ T lymphocytes (inhibiting their cytotoxic effects and cytokine production), and B lymphocytes (inhibiting the production of antibodies by these cells) to promote the activation of regulatory T lymphocytes and inhibit the activation of DCs [16].

It is of utmost importance that MSCs isolated from different tissues, especially those isolated from less invasive sources, are characterized and classified. Additionally, little is known about the effects of proinflammatory stimulation with IFN-*γ* on the biological properties of MSCs. Since our group works with bone tissue engineering applications for the reconstruction of the alveolar bone in cleft lip and palate patients, this study investigated the effects of proinflammatory stimulation with IFN-*γ* on the biological properties of SHED and OOMDSCs. These sources of MSCs are considered noninvasive for cleft lip and palate patients since small fragments of the orbicularis oris muscle are regularly discarded during cheiloplasty surgery [10], and all children have deciduous teeth in exfoliation when they are between six and twelve years old.

The main objective of this work was to study how both OOMDSCs and SHED behave when treated with an inflammatory IFN-*γ* stimulus, specifically regarding their proliferative, osteogenic, and immunomodulatory potentials. Specifically, this study was aimed at determining whether the proliferative capacities of SHED and OOMDSCs are influenced by treatment with IFN-*γ* at different concentrations to evaluate the effect of these treatments on the osteogenic potential of OOMDSCs and SHED by assessing the expression of specific costimulatory, immunogenic, and immunomodulatory molecules in populations of both OOMDSCs and SHED exposed to a proinflammatory IFN-*γ* stimulus or left unexposed.

## 2. Material and Methods

### 2.1. Isolation and Culture of SHED and OOMDSCs

For the establishment of primary lines of MSCs, pulps of deciduous teeth and fragments of the orbicularis oris muscle were obtained from the care service for patients with cleft lip and palate at Hospital Municipal Infantil Menino Jesus. SHED and OOMDSCs isolated from five distinct donors each were isolated and used for the experiments described. For the isolation of SHED, tooth pulps were collected by surgical extraction and immediately added to a sterile collector containing 2 mL of Dulbecco's modified Eagle medium/Nutrient Mixture F-12 (DMEM-F12; Gibco Invitrogen, Grand Island, NY) supplemented with 100 IU/mL penicillin and streptomycin (Penicillin-Streptomycin; Gibco Invitrogen, Grand Island, NY). The pulps of deciduous teeth were then washed with 1X phosphate-buffered saline (PBS^−^, pH: 7.4; Gibco Invitrogen, Grand Island, NY) twice and digested with 1 mg/mL trypsin solution (TrypLE; Gibco Invitrogen, Grand Island, NY) diluted in 1XPBS^−^ (pH 7.4; Gibco Invitrogen, Grand Island, NY) for 30 minutes at 37°C. After tissue digestion, the samples were centrifuged at 300 x g for 5 minutes. Subsequently, the pulps were cut into two or more fragments, depending on size, and cultured in a 12-well plate, preferably with one fragment per well. Twenty days after this procedure, MSCs were expelled from the dental pulp fragments, thereby establishing a primary culture of SHED. In addition, orbicularis oris muscle fragments were collected during cheiloplasty surgery and immediately added to a sterile collector containing 2 mL of DMEM-F12 (Gibco Invitrogen, Grand Island, NY) supplemented with 100 IU/mL of penicillin and streptomycin (Penicillin-Streptomycin; Gibco Invitrogen, Grand Island, NY). The orbicularis oris muscle fragments were then washed twice with 1XPBS^−^ (pH 7.4; Gibco Invitrogen, Grand Island, NY) and digested in a solution containing 1 mg/mL trypsin (TrypLE-Gibco Invitrogen, Grand Island, NY) diluted in 1XPBS^−^ for 30 minutes at 37°C. After enzymatic digestion, the samples were centrifuged at 300 x g for 5 minutes. The muscle fragments were then cut into three or four parts, depending on size, and cultured in 12-well plates, preferably with one fragment per well. Twenty days after this procedure, MSCs were expelled from the orbicularis oris muscle fragments, and a culture of OOMDSCs was established.

The isolated SHED and OOMDSCs were cultured at 37°C/5% CO_2_ in humidified incubators in T25 or T75 flasks in 5 (for T25 flasks) or 10 (for T75 flasks) mL of DMEM-F12 (Gibco Invitrogen, Grand Island, NY) supplemented with 15% fetal bovine serum (FBS; HyClone-GE Heathcare Life Sciences, South Logan, Utah). The culture medium was replaced every 2 or 3 days. The cultured MSCs were transferred to new flasks when approximately 90% confluent to prevent cellular senescence. To this end, the basal medium was aspirated, and the cell culture flasks were washed twice with 5 mL of 1XPBS^−^to remove all the residual culture medium. Thereafter, the MSCs that adhered to the cell culture flasks were removed from the flasks by incubating the MSCs at 37°C/5% CO_2_ for 3 minutes in 1 (for T25 flasks) or 1.5 (for T75 flasks) mL of TrypLE Express Enzyme (1X) (Gibco Invitrogen, Grand Island, NY). After 3 minutes, 3 mL of basal medium was added to the cell culture flasks to neutralize the action of TrypLE Express Enzyme. The cell culture flasks were then washed several times with the basal medium to remove all residual MSCs from the flasks, and the MSC-containing solution was transferred to 15 mL polypropylene tubes (BD Falcon, Heidelberg, Germany). These tubes were subsequently centrifuged at 300 x g for 5 minutes, and the supernatant was aspirated to remove all TrypLE Express Enzyme present in the supernatant. Then, the precipitate containing the MSCs was resuspended in 1 mL of basal medium, and the cells were transferred to new cell culture flasks containing 5 (for T25 flasks) or 10 (for T75 flasks) mL of basal culture medium at a density of 5,000 cells/cm^2^ and kept in culture at 37°C/5% CO_2_ in humidified incubators. For long-term storage, MSCs were maintained in liquid nitrogen. When necessary, MSCs were thawed and expanded to conduct experiments. All experiments described here were performed according to national and international standards of research ethics and were approved by the Research Ethics Committee of Hospital Sirio Libanes.

### 2.2. Cell Surface Marker Analysis

To confirm their identities as MSCs, both SHED and OOMDSCs were characterized by flow cytometry for the expression of typical surface markers of MSCs (CD29, CD44, CD90, CD73, CD105, and CD106) and endothelial (CD31) and hematopoietic (C34) cells. All cells were incubated with antibodies (conjugated with fluorochromes) that had the ability to bind specifically to intracellular and cell surface proteins to compare and characterize the cells according to the expression of specific antigens. A total of 1 × 10^6^ cells obtained from cell cultures and diluted in 100 *μ*L of 1XPBS^−^ were transferred to flow cytometry tubes and incubated with the following monoclonal antibodies for 15 minutes at room temperature in the dark for staining: anti-CD29-PE, anti-CD44-PE, anti-CD73-FITC, anti-CD90-FITC, anti-CD105-PE, anti-CD166-PE, anti-CD34-FITC, and anti-CD31-FITC (BD Bioscience, Becton Dickinson Franklin Lakes, NJ). The samples were then washed with 1XPBS^−^, resuspended in 500 *μ*L of 1XPBS^−^, run on a FACSCalibur (BD, Becton Dickinson, Franklin Lakes, NJ) flow cytometer, and subsequently analyzed using FlowJo software (TreeStar Inc.). A sample of unstained cells was prepared for each experiment to eliminate the influence of any nonspecific staining and innate autofluorescence of the cells. As a negative control for the reactions, an isotype control was used for each antibody.

### 2.3. Osteogenic Differentiation Assay

In addition to being characterized by the expression of cell surface markers, MSCs were also characterized by their potentials for osteogenic, adipogenic and chondrogenic differentiation. For the osteogenic differentiation assays, a total of 5 × 10^3^ MSCs were seeded into 12-well cell culture plates (Corning® Costar®). The MSCs were then allowed to adhere to the surface of the culture plates for 24 hours in basal medium at 37°C/5% CO_2_ in humidified incubators prior to the initiation of the osteogenic differentiation protocol. Osteogenesis was then induced by replacing the basal medium with culture medium containing growth factors specific for the induction of osteogenic differentiation in MSCs (StemPro Osteogenesis Differentiation Kit; Gibco Invitrogen, Grand Island, NY). The osteogenic medium was changed every 3-4 days for 21 days, and osteogenic differentiation was observed during this period by assessing the morphological alteration of spindle-shaped cells into star-shaped cubic cells. The MSCs used as negative controls for the osteogenic differentiation process were cultured in the basal medium for the same 21 days. To evaluate the osteogenic differentiation process, the culture of MSCs was stained with Alizarin Red S after 21 days under the differentiation conditions. The staining of the culture of MSCs with Alizarin Red S indicated the presence of a mineralized matrix in the culture and suggested the presence of calcium hydroxyapatite, indicating that a successful osteogenic differentiation process occurred.

### 2.4. Adipogenic Differentiation Assay

For the characterization of MSCs regarding their adipogenic differentiation potential, a total of 5 × 10^3^ MSCs were seeded into 12-well cell culture plates (Corning® Costar®). The MSCs were then allowed to adhere to the surface of the culture plates for 24 hours in basal medium at 37°C/5% CO_2_ in humidified incubators prior to the initiation of the adipogenic differentiation protocol. Adipogenesis was then induced by replacing the basal medium with culture medium containing specific growth factors to induce adipogenic differentiation in the MSCs (StemPro Adipogenesis Differentiation Kit; Gibco Invitrogen, Grand Island, NY). The adipogenic medium was changed every 3-4 days for 18 days, and adipogenic differentiation was observed by evaluating the presence of vacuoles in the MSCs. The MSCs used as negative controls for the adipogenic differentiation process were cultured in the basal medium for the same 18 days. To evaluate the adipogenic differentiation process, the MSC culture was stained with Oil Red O (Sigma-Aldrich, St. Louis, MO) after 18 days of culture under the differentiation conditions. Oil Red O staining indicated the presence of lipid-rich vacuoles within the MSCs.

### 2.5. Chondrogenic Differentiation Assay

MSCs were also characterized for their chondrogenic differentiation potential. To assess this potential, a total of 5 × 10^3^ MSCs were seeded in 12-well cell culture plates (Corning® Costar®). The MSCs were then allowed to adhere to the surface of the culture plates for 24 hours in basal medium at 37°C/5% CO_2_ in humidified incubators prior to the initiation of the chondrogenic differentiation protocol. Chondrogenesis was induced by replacing the basal medium with culture medium containing growth factors specific for the induction of chondrogenic differentiation in the MSCs (StemPro® Chondrogenesis Differentiation Kit; Gibco Invitrogen, Grand Island, NY) once the MSC culture had reached at least 80% confluency. The chondrogenic medium was changed every 3-4 days for 21 days. The MSCs used as a negative control for the process of chondrogenic differentiation were cultured in the basal medium for the same 21 days. To evaluate the process of chondrogenic differentiation, the culture of MSCs was stained with Alcian Blue (Sigma-Aldrich, St. Louis, MO) after 21 days of culture under the differentiation conditions. The blue color observed by Alcian Blue staining indicated the presence of proteoglycans (extracellular matrix) secreted by chondrocytes and showed that successful chondrogenic differentiation occurred.

### 2.6. Analysis of the Effect of Proinflammatory Stimulation with IFN-*γ* on the Proliferation and Viability of SHED and OOMDSCs

To evaluate the effect of a proinflammatory IFN-*γ* stimulus on the cell proliferation of SHED and OOMDSCs, both cell types were seeded in 12-well plates and maintained in culture medium with or without IFN-*γ* at different concentrations. Initially, a total of 5 × 10^3^ cells were plated in each well of the 12-well plates in culture medium with or without IFN-*γ* at 10, 25, 50, 100, or 500 ng/mL for 3, 5, or 7 days. After the preestablished time for each culture was reached, analysis of cell proliferation and viability during this period was performed by determining the percentage of viable cells and the total number of cells present in each well. For this purpose, both SHED and OOMDSCs were removed from each well of the 12-well plates used, washed, and resuspended in 300 *μ*L of culture medium. Then, 10 *μ*L of MSC-containing solution was diluted in 10 *μ*L of Trypan Blue (Sigma-Aldrich) so that the dead cells could be identified. Finally, the counting process and determination of viability were performed using a Cell Countess (Sigma-Aldrich).

### 2.7. Analysis of the Effect of the Proinflammatory IFN-*γ* Stimulus on the Osteogenic Potential of SHED and OOMDSCs

To study the proinflammatory effect of the IFN-*γ* stimulus on the osteogenic differentiation potential of SHED and OOMDSCs, both cell types were seeded in 96-well plates. A total of 3 × 10^3^ MSCs were seeded in each well and cultured in culture medium with or without IFN-*γ* at different concentrations (10, 25, 50, 100, or 500 ng/mL) for 21 days. After 21 days of differentiation, the wells were stained with Alizarin Red as previously described. Subsequently, 100 *μ*L of 20% methanol solution and 10% acetic acid diluted in 1XPBS^−^ were added to each well so that the mineralized matrix previously stained with Alizarin Red could be dissolved. Finally, the samples were incubated for 15 minutes at room temperature exposed to light, and the osteogenic differentiation process was quantified by determining the optical density (OD) of the solution in each well with a spectrophotometer at a 480 nm wavelength.

### 2.8. Analysis of the Effect of the Proinflammatory IFN-*γ* Stimulus on the Expression of Cell Surface and Intracellular Markers

To study, by flow cytometry, the expression of costimulatory, immunogenic, and immunomodulatory molecules on the cell surface of both OOMDSCs and SHED exposed to a proinflammatory stimulus, both cell types were maintained in T75 flasks in culture medium supplemented with 25 ng/mL IFN-*γ* (PeproTech) for 48 hours. In addition, untreated cells were maintained in culture medium and used as a control for the experiment. After 48 hours, the stem cells maintained in the medium supplemented with IFN-*γ* were extracted from their flasks, processed into a single-cell suspension, and prepared for flow cytometric analysis. This technique was used to evaluate the immunological profile of both the OOMDSCs and the SHED maintained in the medium supplemented with 25 ng/mL IFN-*γ* or no IFN-*γ*. The immunological profiles of both the OOMDSCs and the SHED were assessed by analyzing the expression of CD40, CD80, CD86, human leukocyte antigen- (HLA-) DR, HLA-A, HLA-B, HLA-C, and HLA-G in cells maintained in the medium supplemented with IFN-*γ* or without IFN-*γ*. For the detection of nuclear or cytoplasmic proteins, the cells were fixed and permeabilized to allow the antibodies to cross the cell membrane. A sample of unlabeled cells was prepared for each experimental group to eliminate the influence of nonspecific staining and innate autofluorescence on the results. As a negative control for the reactions, an isotype control was used for each type of immunoglobulin used. The analysis of the expression of the different markers studied (CD40, CD80, CD86, HLA-DR, HLA-A, HLA-B, HLA-C, and HLA-G) in both the SHED and the OOMDSCs treated with IFN-*γ* or left unstimulated was performed by determining the median fluorescence intensity (MFI) of each marker.

### 2.9. Statistical Analysis

Descriptive analyses for quantitative data were performed and are presented as the average accompanied by the corresponding standard deviation (±sd). The assumptions of a normal distribution and homogeneity of the variances were evaluated with the Shapiro-Wilk test and the Levene test, respectively. To analyze two distinct factors, two-way ANOVA was used. For one factor analysis, one-way ANOVA was used. When it was necessary to perform multiple comparisons of means, the Bonferroni post hoc test was used. For comparisons of means between two independent groups, an unpaired Student *t*-test was used. All analyses were performed with the software SigmaPlot for Windows version 11.0 with a significance level of *α* = 0.05.

## 3. Results

### 3.1. SHED and OOMDSCs Express a Typical MSC Immunophenotype and Are Capable of Adipogenic, Osteogenic, and Chondrogenic Differentiation

**Figure 1:**
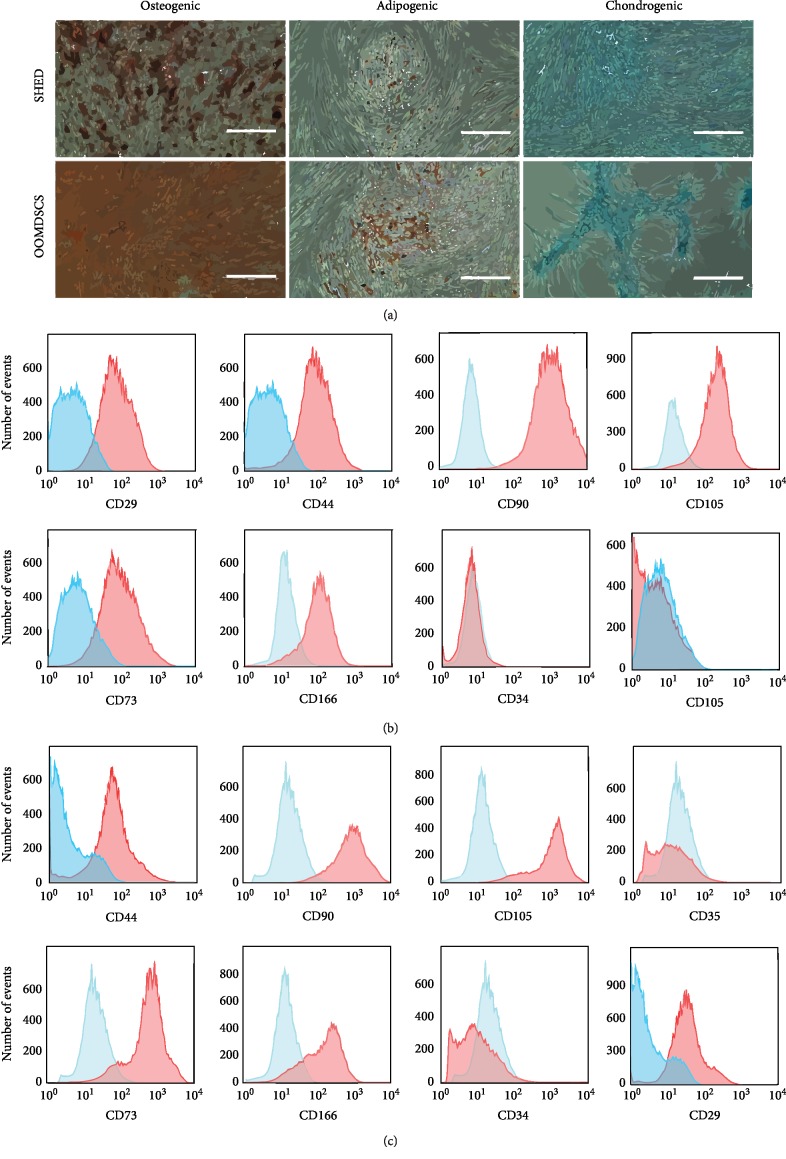
(a) Representative figure of the osteogenic, adipogenic, and chondrogenic differentiation potentials of SHED and OOMDSCs. Both SHED and OOMDSCs were capable of osteogenic, adipogenic, and chondrogenic differentiation after culturing in differentiation medium. Osteogenesis is shown by the presence of a mineralized matrix containing calcium hydroxyapatite (red staining when stained with Alizarin Red S), adipogenesis is shown by the formation of lipid vesicles (red staining when stained with Oil Red O), and chondrogenesis is shown by the production of proteoglycans (blue staining when stained with Alcian Blue). Scale bars represent 400 *μ*m. (b, c) Representative figure of the immunophenotypic profiles of SHED (b) and OOMDSCs (c). Both SHED and OOMDSCs expressed the mesenchymal stem cell markers CD29, CD44, CD90, CD105, CD73, and CD166 but did not express endothelial (CD31) or hematopoietic (CD34) markers on the cell surface. Histograms demonstrate the binding of conjugated antibodies (in red) and isotype controls (in blue) to surface antigens.

After being maintained under specific conditions of differentiation, populations of both SHED and OOMDSCs were differentiated into adipocytes, chondrocytes, or osteocytes. After 21 days of culture in osteogenic differentiation medium, both the OOMDSCs and the SHED were capable of osteogenic differentiation, as evidenced by the presence of a mineralized matrix detected by Alizarin Red S staining. Similarly, both the OOMDSCs and the SHED, when cultured in chondrogenic differentiation medium, were capable of chondrogenic differentiation after 21 days, as demonstrated by positivity with Alcian Blue staining. Finally, both the SHED and the OOMDSCs were able to differentiate into adipocytes after 18 days of culture in adipogenic differentiation medium, as demonstrated by the presence of lipid vacuoles stained with Oil Red O (Figure 1(a)). In addition, flow cytometric analysis revealed that the population of both SHED (Figure 1(b)) and OOMDSCs (Figure 1(c)) was positive for the mesenchymal stem markers CD29, CD44, CD90, CD105, CD73, and CD166 and negative for endothelial (CD31) and hematopoietic cell markers (CD34) (Table 1).

### 3.2. The Osteogenic Differentiation Potential of both SHED and OOMDSCs Was Maintained after Treatment with IFN-*γ*

**Figure 2:**
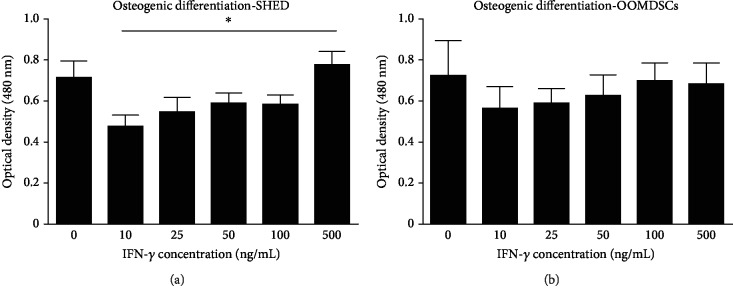
Effects of proinflammatory stimulation with IFN-*γ* on the osteogenic differentiation potential of SHED and OOMDSCs. (a, b) The addition of IFN-*γ* at a concentration of 10 ng/mL, 25 ng/mL, 50 ng/mL, 100 ng/mL, or 500 ng/mL maintained the osteogenic differentiation potential of both SHED (a) and OOMDSCs (b) at levels similar to the level observed with control treatment. A statistically significant difference was observed only between SHED maintained in osteogenic differentiation medium supplemented with IFN-*γ* at a concentration of 500 ng/mL (0.77 ± 0.06 sd) and SHED cultured in osteogenic differentiation medium supplemented with IFN-*γ* at a concentration of 10 ng/mL (0.47 ± 0.06 sd). ^∗^*p* < 0.05, determined by one-way ANOVA followed by the Bonferroni post hoc test. Five experimental replicates were performed for each group for both SHED and OOMDSCs.

Alizarin Red S staining after 21 days of osteogenic differentiation in osteogenic differentiation medium with or without IFN-*γ* at different concentrations (10 ng/mL, 25 ng/mL, 50 ng/mL, 100 ng/mL, and 500 ng/mL) demonstrated that both SHED (Figure 2(a)) and OOMDSCs (Figure 2(b)) maintained their osteogenic differentiation potential when stimulated with IFN-*γ* at all concentrations compared with no stimulation. In particular, SHED had their osteogenic differentiation potential significantly stimulated by the addition of 500 ng/mL IFN-*γ* to the osteogenic differentiation medium compared with the addition of 10 ng/mL IFN-*γ* (Figure 2(a)).

### 3.3. The Proliferation of both SHED and OOMDSCs Was Inhibited after Treatment with IFN-*γ*

**Figure 3:**
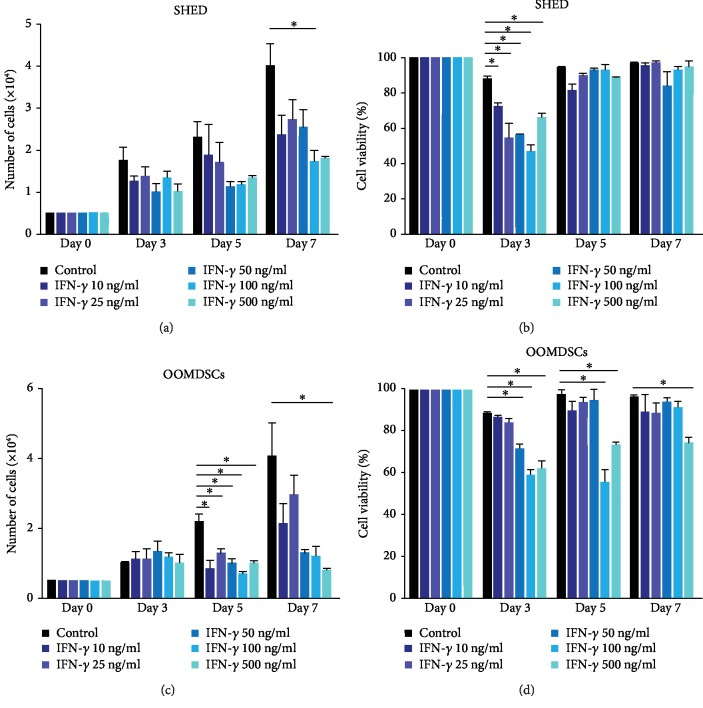
Effects of IFN-*γ* on the proliferative capacity and cell viability of SHED and OOMDSCs. (a) An inhibition of cell proliferation in SHED was observed after three days of culture in culture medium supplemented with IFN-*γ* at different concentrations. After the fifth day of culture, it was observed that the addition of IFN-*γ* at higher concentrations (100 ng/mL and 500 ng/mL) to the culture medium resulted in a greater inhibition of the proliferation of SHED than the addition of IFN-*γ* at lower concentrations (10 ng/mL, 25 ng/mL, and 50 ng/mL); however, this difference was not statistically significant. A similar effect was observed after seven days of cell culture; however, a statistically significant reduction in the cell proliferation of SHED treated with IFN-*γ* at the 100 ng/mL concentration compared with that of untreated SHED was observed. (c) An inhibition of proliferation in OOMDSCs was observed after five days of culture in culture medium supplemented with IFN-*γ* at different concentrations. Beginning on the fifth day of culture, it was observed that compared with no IFN-*γ* addition, the addition of IFN-*γ* to the culture medium resulted in a statistically significant inhibition of OOMDSC proliferation. After seven days of culture, the addition of IFN-*γ* at higher concentrations (50 ng/mL, 100 ng/mL, and 500 ng/mL) to the culture medium resulted in a greater inhibition of OOMDSC proliferation; however, compared with the OOMDSCs maintained in culture medium without IFN-*γ*, only the OOMDSCs treated with IFN-*γ* at 500 ng/mL had their proliferation significantly inhibited. (b) A statistically significant decrease in the cell viability of SHED was observed only on the third day of culture in culture medium supplemented with IFN-*γ* at distinct concentrations. Furthermore, a greater decrease in the viability of SHED was detected in the groups treated with IFN-*γ* at a 25 ng/mL, 50 ng/mL, or 100 ng/mL concentration than in the groups treated with lower concentrations of IFN-*γ*. No significant differences were observed among the populations of SHED after five or seven days of culture in culture medium supplemented with IFN-*γ* at distinct concentrations. (d) For OOMDSC populations, a statistically significant decrease in cell viability on the third day of culture was observed only when the cells were treated with IFN-*γ* at 50 ng/mL, 100 ng/mL, or 500 ng/mL. On the fifth day of culture, however, a statistically significant decrease in cell viability was observed only when OOMDSCs were treated with IFN-*γ* at 100 ng/mL or 500 ng/mL. Additionally, a statistically significant decrease in cell viability was observed on the seventh day of culture only when OOMDSC populations were treated with IFN-*γ* at a concentration of 500 ng/mL. ^∗^*p* < 0.05, determined by two-way ANOVA followed by the Bonferroni post hoc test. Five experimental replicates were performed for each group for both SHED and OOMDSCs.

The results obtained in this study demonstrated that, regarding their proliferative capacity and cell viability, populations of both SHED and OOMDSCs behaved in a similar manner when treated with a proinflammatory IFN-*γ* stimulus. After the third day of culture, compared with no treatment, the addition of distinct concentrations of IFN-*γ* to the culture medium resulted in the inhibition of cell proliferation in both SHED (Figure 3(a)) and OOMDSCs (Figure 3(c)). The addition of IFN-*γ* to the culture medium significantly inhibited the proliferation of SHED and OOMDSCs during all days of culture, and this inhibition was more evident in the final days of culture (days 5 and 7) than in the initial days. Furthermore, the addition of IFN-*γ* at higher concentrations (100 ng/mL and 500 ng/mL) more strongly inhibited the proliferation of both SHED and OOMDSCs than the addition of IFN-*γ* at lower concentrations (10 ng/mL, 25 ng/mL, and 50 ng/mL). In addition, a significant decrease in the viability of both SHED (Figure 3(b)) and OOMDSCs (Figure 3(d)) was mainly observed in the initial days of culture and was more evident when the stem cells were treated with the higher concentrations of IFN-*γ*. After seven days of culture, the viability of most of the IFN-*γ*-treated populations of both SHED and OOMDSCs returned to levels similar to those of the untreated controls. Due to the fact that IFN-*γ* was only added to the culture medium in the beginning of the experiment, it can be hypothesized that the increase in cell viability observed is a result of IFN-*γ* losing its biological function or being depleted from the culture medium after the third day of culture.

### 3.4. SHED and OOMDSCs Expressed Molecules with Immunomodulatory Properties but Did Not Express Costimulatory Molecules or HLA Class II Molecules after Treatment with IFN-*γ*

**Figure 4:**
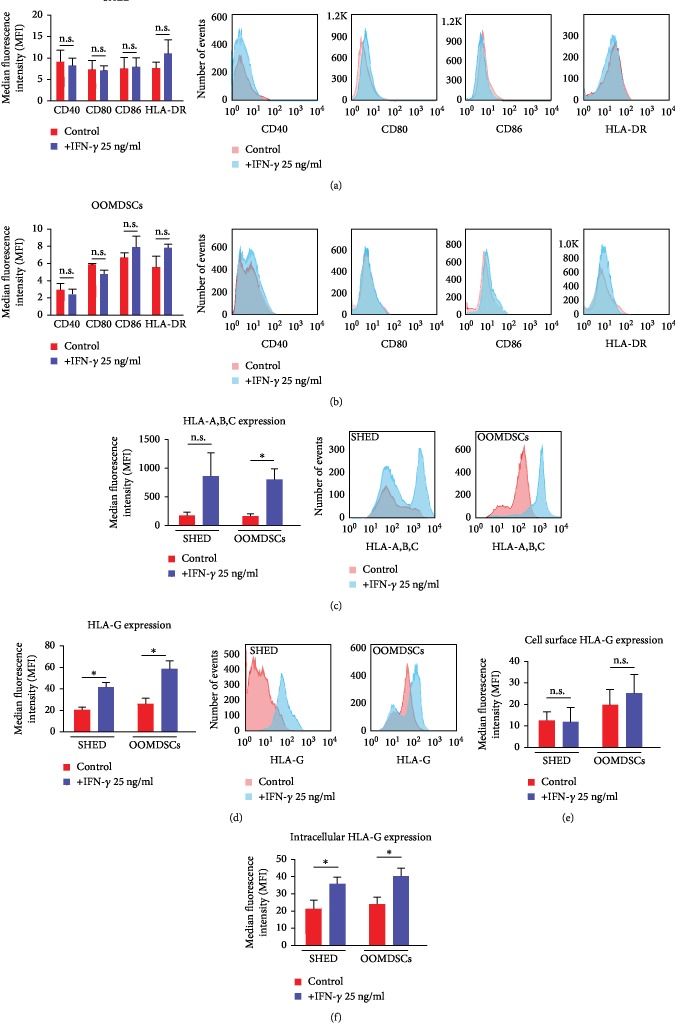
Effects of IFN-*γ* on the expression of immunogenic, costimulatory, and immunomodulatory molecules on the cell surface of SHED and OOMDSCs. (a, b) Neither SHED (a) nor OOMDSCs (b) expressed the costimulatory molecules CD40, CD80, and CD86 or the human leukocyte antigen class II HLA-DR on their cell surface, either in the absence of proinflammatory stimulation with IFN-*γ* or when stimulated with 25 ng/mL IFN-*γ*. (c) Higher expression of the human leukocyte antigen class I molecules HLA-A, HLA-B, and HLA-C was observed on the cell surface of both SHED and OOMDSCs treated with 25 ng/mL IFN-*γ* than on that of MSCs that were not treated with IFN-*γ*. However, this increase in the MFI value was statistically significant only when the OOMDSCs were treated with 25 ng/mL IFN-*γ*. (d) After treatment with 25 ng/mL IFN-*γ*, the expression of the immunomodulatory molecule HLA-G by both OOMDSCs and SHED was higher than that in the corresponding control-treated cells. This increase in the MFI value was statistically significant for both the SHED and the OOMDSCs treated with 25 ng/mL IFN-*γ*. (e, f) The effects of IFN-*γ* on the cell surface and intracellular expression of HLA-G were assessed. (e) Cell surface HLA-G expression remained unchanged in SHED treated with 25 ng/mL IFN-*γ* but increased in OOMDSCs treated with 25 ng/mL IFN-*γ*; however, this increase was not statistically significant. (f) Intracellular HLA-G expression increased significantly in both SHED and OOMDSCs treated with 25 ng/mL IFN-*γ*. Histograms demonstrate the binding of conjugated antibodies (in red) and isotype controls (in blue) to antigens. ^∗^*p* < 0.05, determined by an unpaired Student *t*-test. Five experimental replicates were performed for each group for both SHED and OOMDSCs.

After 48 hours of proinflammatory stimulation with 25 ng/mL IFN-*γ*, flow cytometric analysis demonstrated that both SHED and OOMDSCs did not express HLA-DR (SHED = 10.95 ± 3.38 MFI, OOMDSCs = 7.78 ± 0.5 MFI) or the costimulatory molecules CD40 (SHED = 8.26 ± 1.78 MFI, OOMDSCs = 2.42 ± 0.64 MFI), CD80 (SHED = 7.03 ± 1.17 MFI, OOMDSCs = 4.74 ± 0.52 MFI), and CD86 (SHED = 7.82 ± 2.27 MFI, OOMDSCs = 7.85 ± 1.32 MFI) on their cell surface, and these expression levels were similar to the expression levels of HLA-DR (SHED = 7.53 ± 1.47 MFI, OOMDSCs = 5.54 ± 1.34 MFI), CD40 (SHED = 9.1 ± 2.79 MFI, OOMDSCs = 2.97 ± 0.780 MFI), CD80 (SHED = 7.18 ± 2.28 MFI, OOMDSCs = 5.87 ± 0.19 MFI), and CD86 (SHED = 7.41 ± 2.77 MFI, OOMDSCs = 6.64 ± 0.58 MFI) observed in untreated controls (Figures 4(a) and 4(b)). However, the expression of HLA-A, HLA-B, and HLA-C was detected in the untreated populations of both SHED (172.36 ± 58.94 MFI) and OOMDSCs (159.96 ± 39.16 MFI), and after treatment with IFN-*γ*, the HLA-A,HLA-B, and HLA-C expression on the cell surface of both SHED (858.8 ± 413.63 MFI) and OOMDSCs (788.8 ± 191.7 MFI) increased (Figure 4(c)). Additionally, the expression of the immunomodulatory molecule HLA-G was detected in the unstimulated populations of both SHED (20.34 ± 2.46 MFI) and OOMDSCs (25.89 ± 4.74 MFI). After treatment with 25 ng/mL IFN-*γ*, HLA-G expression increased significantly in both SHED (41.08 ± 4.40 MF) and OOMDSCs (58.18 ± 7.45 MFI) (Figure 4(d)). Finally, the intracellular and cell surface expression of HLA-G was assessed in both SHED and OOMDSCs treated with or without 25 ng/mL IFN-*γ* to verify whether the increase in HLA-G expression observed previously after treatment with 25 ng/mL IFN-*γ* was due to increased HLA-G surface or intracellular expression. The results demonstrated that while the intracellular expression of HLA-G increased significantly in the populations of both SHED (control = 21.5 ± 4.82 MFI, 25 ng/mL IFN‐*γ* = 35.93 ± 3.81 MFI) and OOMDSCs (control = 24.2 ± 3.74 MFI, 25 ng/mL IFN‐*γ* = 40.25 ± 4.74 MFI) treated with 25 ng/mL IFN-*γ* (Figure 4(f)), the expression of HLA-G on the cell surface of SHED treated with 25 ng/mL IFN-*γ* did not change (control = 12.90 ± 4.09 MFI, IFN‐*γ* 25 ng/mL = 12.21 ± 6.39 MFI), but the expression of HLA-G on the cell surface of OOMDSCs increased when these cells were treated with 25 ng/mL IFN-*γ* (control = 20.15 ± 6.91 MFI, 25 ng/mL IFN‐*γ* = 25.48 ± 8.79 MFI); however, this increase was not statistically significant (Figure 4(e)).

## 4. Discussion

The results obtained in the present study demonstrated that SHED and OOMDSCs behave in a similar way when considering the effect of a proinflammatory IFN-*γ* stimulus on proliferative capacity. The proliferation of both SHED and OOMDSCs was inhibited after the addition of IFN-*γ* to the medium used for culture. In addition, treatment with IFN-*γ* at higher concentrations resulted in greater inhibition of the proliferation of both SHED and OOMDSCs than treatment with IFN-*γ* at lower concentrations. However, varied results have been reported in relation to the effect of proinflammatory stimulation on the proliferative capacity of MSCs. For example, in a study by He et al. [27], it was shown that treatment of dental pulp stem cells with IFN-*γ* at low concentrations (0.05, 0.5, and 5 ng/mL) stimulated the proliferation and migration of this type of stem cell. On the other hand, Chan et al. [28] demonstrated that compared with untreated MSCs, MSCs treated with IFN-*γ* for 8 days had their proliferative capacity reduced by 50%. Similarly, Yazid et al. [29] demonstrated that MSCs isolated from healthy dental pulp have a greater proliferative capacity than MSCs isolated from inflamed dental pulp. In addition, in a study by Qin et al. [30], no statistically significant differences in the proliferative capacity of dental pulp stem cells were reported when these stem cells were treated with the proinflammatory cytokine TNF-*α*, regardless of the concentration of the cytokine in the culture medium. Regarding the possible mechanism underlying the stimulation of proliferation in MSCs after treatment with IFN-*γ*, He et al. [27] demonstrated the occurrence of increases in PCNA and Ki-67 expression after IFN-*γ* treatment, indicating a high percentage of cells undergoing division. This study further demonstrated that the expression of cell cycle-promoting proteins such as Cyclin B1, Cyclin D1, and PCNA was increased, whereas the expression of proteins that inhibit cell cycle progression (such as P21) was reduced after stimulation of MSCs with IFN-*γ* [27]. Furthermore, Qin et al. [30] demonstrated that TNF-*α* was capable of stimulating the proliferation of human dental pulp stem cells through the Akt/Glycogen Synthase Kinase-3*β*/Cyclin D1 signaling pathways. Mechanisms related to the inhibition of proliferation in MSCs have also been observed in some studies after treatment of MSCs with proinflammatory cytokines. A study conducted by Croitoru-Lamoury et al. [31], for example, demonstrated that IFN-*γ* treatment was capable of activating indolamine-2,3-dioxygenase (IDO) in human and murine MSCs and that the activation of IDO could inhibit the proliferation of MSCs through the depletion of tryptophan, an essential amino acid required for protein biosynthesis. In addition, the production of tryptophan metabolites (e.g., kynurenine, 3-hydroxyanthranilic acid and quinolinic acid) by MSCs might be able, by a negative feedback mechanism, to inhibit the proliferation of these stem cells.

The present study also demonstrated that the osteogenic differentiation potential of populations of both SHED and OOMDSCs was maintained when these cells underwent a proinflammatory stimulation with IFN-*γ*. However, a study conducted by He et al. [27] demonstrated that low concentrations of the proinflammatory cytokine IFN-*γ* can inhibit the odontogenic and osteogenic differentiation of dental pulp stem cells through the nuclear factor kappa B (NF-*κ*B) (p65) and MAPK (P38) signaling pathways. Sonoda et al. [26] also demonstrated that dental pulp stem cells isolated from teeth with irreversible pulpitis and treated with 100 ng/mL IFN-*γ* showed increased expression of osteoblast-specific genes, such as Runx2, alkaline phosphatase, and osteocalcin, after proinflammatory stimulation with IFN-*γ*. The effects of the addition of proinflammatory and immunomodulatory cytokines on the osteogenic differentiation potential of MSCs have also been reported in recent studies. A study conducted by Liu et al. [25] demonstrated that MSC-mediated bone regeneration was partially controlled by the host's local microenvironment and that the action of the host's immune cells and the production of inflammatory cytokines by host cells could considerably affect this process. This study showed that the osteogenic potential of autologous MSCs was adversely affected when these cells were transplanted into wild-type mice, while abundant bone formation was observed when the MSCs were transplanted into immunosuppressed mice.

In addition, studies conducted by Feng et al. [32] and Xing et al. [33] demonstrated that the addition of IGF-1 and TNF-*α* stimulated the osteogenic differentiation potential of dental pulp stem cells through the mammalian target of rapamycin (mTOR) and the NF-*κ*B signaling pathways, respectively. The NF-*κ*B pathway is active in many inflammatory diseases, such as arthritis, gastritis, and pulpitis [34]. Previous studies have shown that NF-*κ*B pathway signaling is involved in the regulation of odontogenic and osteogenic differentiation in dental pulp stem cells [35, 36]. Additionally, the proinflammatory cytokine TNF-*α* can stimulate the odontogenic and osteogenic differentiation of dental pulp stem cells via the NF-*κ*B signaling pathway [32]. A study by He et al. [27] demonstrated that IFN-*γ* positively regulated P65 phosphorylation, which resulted in the inhibition of odontogenic and osteogenic differentiation in dental pulp stem cells, whereas pyrrolidinedithiocarbamate (PDTC), a specific inhibitor of the NF-*κ*B signaling pathway, significantly suppressed the phosphorylation of the p-P65 protein and rescued the odontogenic and osteogenic differentiation capacities of dental pulp stem cells, indicating that the NF-*κ*B pathway plays important roles in the odontogenic and osteogenic differentiation of dental pulp stem cells and that this pathway is regulated by IFN-*γ*. In addition, this study also demonstrated that the NF-*κ*B signaling pathway was not the only pathway involved in the odontogenic and osteogenic differentiation of dental pulp stem cells induced by IFN-*γ* since IFN-*γ* also negatively regulates the phosphorylation of the p38 protein associated with the MAPK signaling pathway. It has been shown that the p38-MAPK signaling pathway is associated with odontoblastic stimulation during tertiary dentinogenesis by p38 phosphorylation and increased nuclear translocation [37]. In addition, TNF-*α* treatment is capable of activating the p38 pathway in dental pulp stem cells via p38 phosphorylation, while p38 inhibition decreases the expression of dentin phosphoprotein (DPP) and dentin sialoprotein (DSP) in these cells [37]. Finally, a study conducted by He et al. [27] showed that lipopolysaccharide (LPS) could promote odontogenic differentiation in human dental pulp stem cells via the MAPK signaling pathway. In addition, this study demonstrated that blockade of the p38-MAPK signaling pathway through the use of a specific inhibitor (SB203580) resulted in a significant rescue of the osteogenic differentiation potential in dental pulp stem cells induced by IFN-*γ*, indicating that the p38-MAPK signaling pathway plays an important role in bone regeneration responses.

The results obtained in this study also demonstrated that both SHED and OOMDSCs do not express HLA-DR or the costimulatory molecules CD40, CD80, and CD86 on their cell surface, even when stimulated with IFN-*γ* at 25 ng/mL for 48 hours. Similarly, a study conducted by Majumdar et al. [38] demonstrated that HLA class II molecules, such as HLA-DR, were not constitutively expressed by MSCs. HLA-DR is expressed by antigen-presenting cells and binds to T cell receptors during the immune response [39]. HLA-DR is not constitutively expressed in MSCs isolated from the bone marrow, but its expression has been shown to be positively regulated after proinflammatory stimulation [40]. However, in this study, we observed that neither SHED nor OOMDSCs expressed HLA-DR on their cell surface and that the expression of this protein remained unchanged in both types of MSCs even after stimulation with 25 ng/mL IFN-*γ* for 48 hours. In contrast, the costimulatory molecules CD40, CD80, and CD86, which are expressed by antigen-presenting cells [41], are not expressed by MSCs isolated from the bone marrow even after proinflammatory stimulation [42]. Therefore, both the SHED and the OOMDSCs used in this study should be incapable of stimulating the activation of lymphocytes, similar to the lack of stimulatory capacity observed in MSCs isolated from the bone marrow [43]. However, it was observed in this study that both SHED and OOMDSCs express the HLA class I molecules HLA-A, HLA-B, and HLA-C on their cell surface before treatment with IFN-*γ*. After 48 hours of proinflammatory stimulation with 25 ng/mL IFN-*γ*, HLA-A, HLA-B, and HLA-C expression on the cell surface of both SHED and OOMDSCs was increased. HLA class I molecules are responsible for the host response to intracellular pathogens and are important for the induction of cellular immunity [44]. A study conducted by Chan et al. [28] demonstrated that treatment of MSCs isolated from the bone marrow of healthy donors with IFN-*γ* increased the expression of HLA class I molecules on the surface of these cells after only 1 day, whereas increased expression of HLA class II molecules could only be observed 4 days after treatment with IFN-*γ*. Therefore, it is possible to hypothesize that the observed difference between the expression of HLA-DR and that of HLA-A, HLA-B, and HLA-C after two days of treatment with IFN-*γ* is due to slower intracellular transport of HLA class II molecules to the cell surface in both SHED and OOMDSCs. However, this hypothesis still needs to be confirmed. In addition, the present study demonstrated that both SHED and OOMDSCs express HLA-G at low levels. The expression of HLA-G increased in both SHED and OOMDSCs treated with 25 ng/mL IFN-*γ* for 48 hours compared with that in the corresponding untreated control cells. In particular, a significant increase in the intracellular expression of HLA-G was detected in both SHED and OOMDSCs after treatment with 25 ng/mL IFN-*γ*.

Similarly, previous studies have demonstrated that MSCs isolated from the bone marrow of baboons expressed HLA class I molecules such as HLA-A, HLA-B, HLA-C, and HLA-G at low levels on their cell surface [43]. HLA-G, in particular, is crucial to the maintenance of immunological tolerance during pregnancy, and its expression is indicative of the occurrence of strong immunosuppression [45]. HLA-G expression may also be upregulated in several tissues under “pathological” conditions, such as cancer [46]. In addition, a study conducted by Carosella et al. [47] reported that HLA-G expression was a prognostic indicator of graft tolerance in patients undergoing heart, kidney, liver, or hematopoietic stem cell transplantation. Phenotypic studies have demonstrated that both fetal and adult MSCs express HLA-G on their cell surface and intracellularly [48]. These studies detected the presence of HLA-G in protein extracts from fetal MSCs, whereas only HLAG mRNA was detected in adult MSCs. HLA-G can be expressed in seven different isoforms, depending on the alternative splicing of the primary transcript, and these isoforms include four membrane proteins (HLA-G1, HLA-G2, HLA-G3, and HLA-G4) and three soluble proteins (HLA-G5, HLA-G6, and HLA-G7) [47]. Regarding the biological activity of the HLA-G molecule, interaction of the HLA-G molecule with the specific HLA-G receptors KIR2DL4 and ILT-2 expressed in NK cells inhibits the adhesive and migratory capacities of NK cells. The expression of HLA-G also inhibits the cytolytic potential of activated CD8^+^ T lymphocytes and stimulates apoptosis in these lymphocytes when they are stimulated with phytohemagglutinin [49, 50]. In addition, the proliferation of allogeneic antigen-induced CD4^+^ T lymphocytes is inhibited by both the soluble isoform HLA-G5 and the membrane-bound isoform HLA-G1 binding to their cognate receptors ILT2 and ILT4 expressed on the cell membrane. The HLA-G1 isoform also prevents the maturation of DCs, rendering them immunosuppressive as a result of the expression of TGF-*β* and IL-10 [51, 52].

The expression of HLA-G molecules by MSCs plays an important role in the immunosuppressive potential of these stem cells. This role has been demonstrated in studies in which the binding of the HLA-G molecule to its receptors was blocked by neutralizing antibodies. As a result, the abilities of MSCs to stimulate the expansion of CD4^+^CD25^+^FoxP3^+^ regulatory T cells inhibit the proliferation of T lymphocytes and suppress the cytotoxic function of NK cells [53, 54]. In addition, a study conducted by Yazid et al. [29] demonstrated that dental pulp stem cells isolated from healthy dental pulp express HLA-G, HLA-A, HLA-B, and HLA-C in higher amounts than dental pulp stem cells isolated from inflamed dental pulp. It was suggested by Chan et al. [28] that upon the binding of IFN-*γ* to its cognate receptors (IFN-*γ*R1 and IFN-*γ*R2), signal transducer and activator of transcription 1 (STAT1) is phosphorylated by JAK1 and JAK2 and undergoes homodimerization. Dimerized STAT1 then translocates into the nucleus and binds to IFN-*γ* activation site (GAS) elements to initiate the transcription of interferon-stimulated genes (ISGs), including interferon-1 regulatory factor (IRF-1). IRF-1, in turn, regulates the transcription of other ISGs via IFN-stimulated response elements (ISRE), leading to the synthesis of HLA class I molecules (such as HLA-G, HLA-A, HLA-B, and HLA-C). Together, IRF-1 and dimerized STAT1 bind to the GAS promoter regions of the gene encoding the CIITA protein to promote gene transcription. The expression of the CIITA protein, on the other hand, induces the expression of HLA class II molecules (such as HLA-DR) [28].

## 5. Final Considerations

It is possible to conclude that SHED and OOMDSCs have their proliferation inhibited and their osteogenic differentiation maintained upon stimulation with IFN-*γ*. We also confirmed that the immunomodulatory potential of these cells is stimulated when they are treated with IFN-*γ*. Thus, our data suggest that both SHED and OOMDSCs are interesting stem cell populations that can be used for clinical applications and that the treatment of these cells with IFN-*γ* can enhance their immunomodulatory potential and further increase their therapeutic potential.

## Figures and Tables

**Table 1:** Average expression of mesenchymal, hematopoietic, and endothelial stem cell markers.

Cell surface marker	Average expression of cell surface markers in SHED (% of positive cells in relation to total)	Average expression of cell surface markers in OOMDSCs (% of positive cells in relation to total)
CD29	91, 3% ± 2, 3%	83, 6% ± 3, 1%
CD44	93, 2% ± 1, 4%	86, 7% ± 2, 7%
CD90	98, 1% ± 0, 6%	94, 8% ± 1, 1%
CD73	90, 2% ± 1, 7%	87, 4% ± 3, 5%
CD105	96, 8% ± 0, 8%	96, 8% ± 0, 5%
CD166	88, 9% ± 4, 1%	71, 8% ± 5, 5%
CD34	0, 42% ± 0, 23%	2, 2% ± 0, 71%
CD31	0, 46% ± 0, 31%	2, 6% ± 0, 84%

## Data Availability

The data used to support the findings of this study are available from the corresponding author upon request.
